# A distributed coding logic for thermosensation and inflammatory pain

**DOI:** 10.1038/s41586-025-08875-6

**Published:** 2025-04-23

**Authors:** Nima Ghitani, Lars J. von Buchholtz, Donald Iain MacDonald, Melanie Falgairolle, Minh Q. Nguyen, Julia A. Licholai, Nicholas J. P. Ryba, Alexander T. Chesler

**Affiliations:** 1 National Center for Complementary and Integrative Health, Bethesda, MD USA; 2 National Institute of Dental and Craniofacial Research, Bethesda, MD USA; 3 National Institute of Neurological Disorders and Stroke, Bethesda, MD USA

**Keywords:** Pain, Ion channels in the nervous system

## Abstract

Somatosensory neurons encode detailed information about touch and temperature and are the peripheral drivers of pain^1,2^. Here by combining functional imaging with multiplexed in situ hybridization^3^, we determined how heat and mechanical stimuli are encoded across neuronal classes and how inflammation transforms this representation to induce heat hypersensitivity, mechanical allodynia and continuing pain. Our data revealed that trigeminal neurons innervating the cheek exhibited complete segregation of responses to gentle touch and heat. By contrast, heat and noxious mechanical stimuli broadly activated nociceptor classes, including cell types proposed to trigger select percepts and behaviours^4–6^. Injection of the inflammatory mediator prostaglandin E2 caused long-lasting activity and thermal sensitization in select classes of nociceptors, providing a cellular basis for continuing inflammatory pain and heat hypersensitivity. We showed that the capsaicin receptor TRPV1 (ref. ^7^) has a central role in heat sensitization but not in spontaneous nociceptor activity. Unexpectedly, the responses to mechanical stimuli were minimally affected by inflammation, suggesting that tactile allodynia results from the continuing firing of nociceptors coincident with touch. Indeed, we have demonstrated that nociceptor activity is both necessary and sufficient for inflammatory tactile allodynia. Together, these findings refine models of sensory coding and discrimination at the cellular and molecular levels, demonstrate that touch and temperature are broadly but differentially encoded across transcriptomically distinct populations of sensory cells and provide insight into how cellular-level responses are reshaped by inflammation to trigger diverse aspects of pain.

## Abstract (web summary)

Functional imaging and multiplexed in situ hybridization were combined to investigate how trigeminal neurons encode heat and mechanical stimuli, revealing distinct cellular mechanisms for continuing pain, heat hypersensitivity and tactile allodynia during inflammation.

## Main

Sensory neurons innervating the skin provide animals with valuable input about their immediate surroundings, including the ability to sense touch and temperature^1,2^. In keeping with their varied roles, the receptor neurons with cell bodies in dorsal root and trigeminal ganglia have diverse peripheral morphology, central projections and biophysical profiles^1,2,8^. They differentially express a wide range of marker genes, including receptors, ion channels and neuropeptides^2,8,9^, and can be divided into about a dozen distinctive transcriptomic classes on the basis of single-cell RNA sequencing^10–13^. One key unanswered question is how distinct natural stimuli encoded across these classes allow us to instantaneously localize and discriminate a world of perceptions, for example, distinguish a drop of rain from an insect crawling on the skin, and elicit emotional responses ranging from pleasure to disgust or pain. Equally important would be to understand how this input is altered by injury or inflammation to trigger localized hypersensitivity, allodynia and continuing pain.

Over the past 30 years, identification of thermosensitive^7,14–16^ and mechanosensory^17,18^ ion channels has established a mechanistic framework for how physical stimuli are transduced to evoke neuronal firing in sensory neurons. These proteins also help explain some aspects of sensory coding but often exhibit complex expression across multiple classes^10–13^ and functional redundancy^16^. Therefore, we applied a platform that we developed for matching in vivo function and transcriptomic class^3^ to decode how heat and naturalistic mechanical stimuli are differentially represented in the trigeminal system, the role of the capsaicin and noxious heat receptor (TRPV1) and how sensory detection is transformed during inflammation to drive pain.

## Peripheral coding of heat

**Fig. 1:**
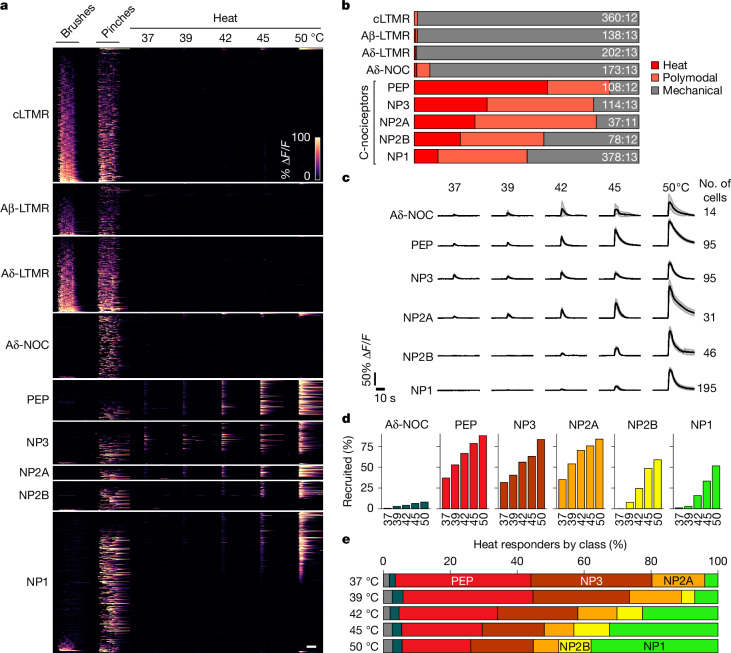
Detection of heat and differential tuning of somatosensory neuron classes. **a**, Heat maps showing in vivo GCaMP responses from 1,588 transcriptomically classified neurons from 15 mice tested with mechanical stimulation and heat. Heat maps are grouped by neuronal class with changes in GCaMP fluorescence (% Δ*F*/*F*) colour-coded as indicated by the scale bar. **b**, Proportions of heat (red), polymodal (orange) and mechanically tuned neurons (grey) for each class; the number of neurons and mice used for assignment are indicated. **c**, Mean responses of heat-responsive nociceptors by class (solid trace; numbers of heat-responsive cells indicated); the shaded area represents 95% confidence interval. **d**, Fractional recruitment of cheek-responsive neurons by class and temperature. **e**, Fractional representation of heat responses by class across the temperature range. Colour coding is as in **d** with LTMRs in grey; numbers of mice and cells are detailed in Supplementary Table 1. Scale bars, 10 s.

**Extended Data Fig. 1:**
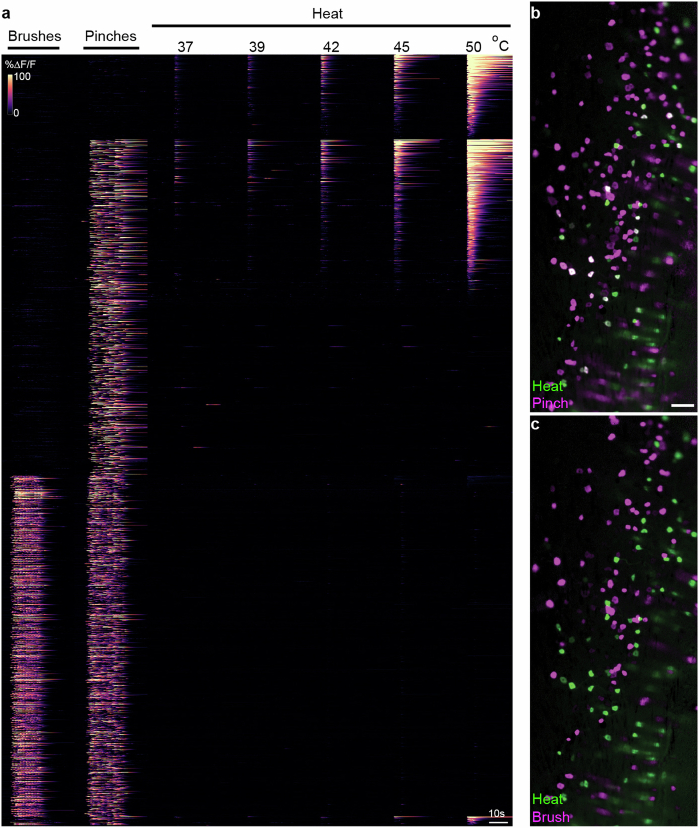
Response properties of trigeminal neurons during mechanical and thermal stimulation of the mouse cheek. a) Heatmap showing the in vivo GCaMP responses from 2085 neurons responding to mechanical and thermal stimulation of the cheek in 15 mice; changes in GCaMP fluorescence (% ΔF/F) are color-coded as indicated in the scale bar. (b, c) Spatial activity map showing the full functional imaging field from an example mouse; cellular response magnitude is indicated by brightness and response to heat (50 °C, green) and (b) pinch or (c) brush responses (magenta). Note significant overlap of heat and noxious mechanical (b) but not gentle touch (c); scale bar: 50 µm.

**Extended Data Fig. 2:**
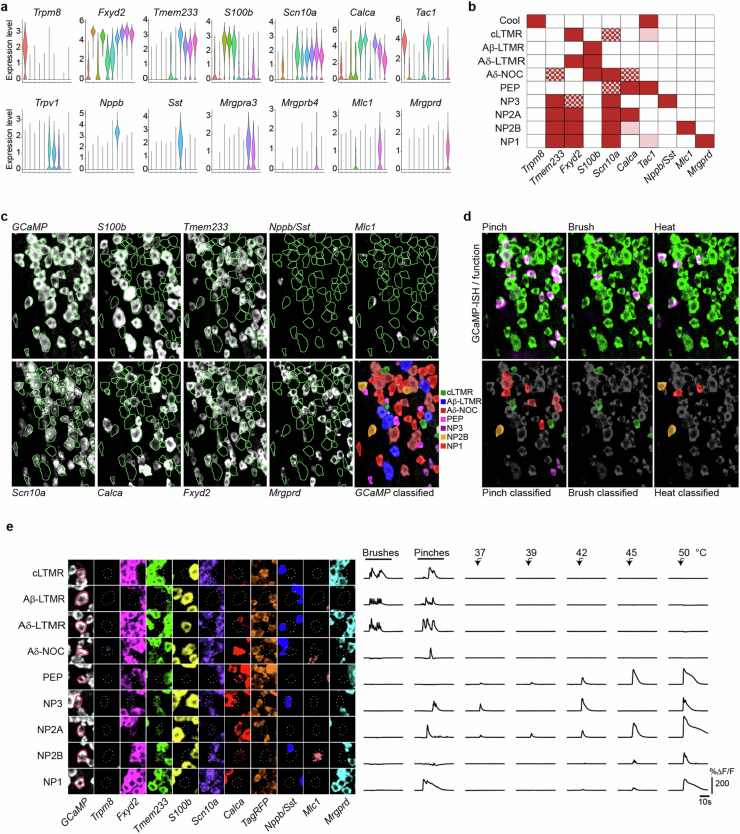
Basis for ISH-based mapping of functional responses to transcriptomic class. a) Violin-plot analysis of expression level (log normalized single cell RNA sequencing data)^11,28^ showing expression patterns of several marker genes used in this study across 10 major transcriptomic classes^10^ in the order shown in (b). At the single cell level, some of these classes can be further divided into subclasses^11,12^ but neither the use of Cre-lines^28^ nor our approach recapitulate this resolution due to lack of markers with appropriate specificity and expression level. (b) Simplified representation of expression of a subset of these markers (red, positive; pink, weak expression; check, not diagnostic; white, negative) defines trigeminal neural classes. (c) Example images of a region of trigeminal ganglion subjected to functional imaging that was aligned to post-hoc multigene ISH. Upper left panel shows ISH for *GCaMP* with positive cells outlined in green. These cell outlines have been transferred to the ISH images for eight genes used in classification to demonstrate the diagnostic power of our approach. The positive and negative expression of these markers allowed unambiguous class assignment for all *GCaMP* expressing cells (lower right panel). Note, the viral approach for *GCaM*P expression (see Methods) results in stochastic *GCaMP* expression in a subset of sensory neurons (approx. 40% in the image shown) accounting for ISH-positive cells that are not *GCaMP* positive and therefore not outlined in green. (d) Upper panels show ISH for *GCaMP* (green) superimposed on the aligned in vivo functional imaging data for pinch, brush and heat (maximum projection images, magenta). Previously^3^, we quantified the fidelity of alignment and showed that although there was not a pixel-to-pixel match between the different imaging modes, <5% of cells were displaced by more than 30 % of their diameter. Lower panels show the responding cells color-coded for transcriptomic class. As expected, each stimulus only activates a small subset of the *GCaMP*-positive trigeminal neurons since we only target stimulation to a small region of the cheek. In this example, the cLTMRs (green) responded to brush and pinch but not heat. Over the complete dataset for this study, in experiments where probes for all classes were assayed, more than 96% of functionally responding cells could be classified unambiguously and less than 2% had an ambiguous pattern that could not be resolved using the classification logic. (e) The red outlined GCaMP expressing neurons (dotted white outlines in the other ISH images) were classified and their GCaMP-transients are shown to the right. For the Ca-traces, ΔF/F and time are indicated by scale bars and arrows above the heat traces indicate the start and duration of heating pulses.

To evaluate how the somatosensory system detects and encodes thermal and mechanical stimuli, we used calcium imaging to record single-cell responses from the trigeminal ganglion while stimulating the cheek^3,19^. Across 15 mice, brush, pinch and defined heat pulses activated hundreds of neurons (Extended Data Fig. 1a), which could be divided into thermally or mechanically selective and polymodal cells (Extended Data Fig. 1b). We next used post hoc multigene in situ hybridization (ISH)^3^ to determine how transcriptomic class governs the function of trigeminal neurons (Extended Data Fig. 2) and matched sensory tuning to ten distinct molecular classes. Data from 1,588 neurons (Fig. 1a) revealed clear segregation of gentle touch and heat responses at both the cellular and transcriptomic-class levels. By contrast, nociceptors were often polymodal^20^ (Fig. 1a,b); nonetheless, their tuning varied according to class (Fig. 1a–d) with very few Aδ-nociceptors (Aδ-NOC) responsive to heat, which instead primarily activated unmyelinated C-fibres.

Many studies have identified peptidergic (PEP) nociceptors that express high levels of the heat- and capsaicin-activated ion channel *Trpv1* and the nerve growth factor receptor *Ntrk1* as canonical detectors of noxious heat^2,8,21–23^. Our data confirmed that this neuronal class is particularly sensitive and selective for heat over pinch, with about half of the cells in this class responding only to temperature (Fig. 1b–e). However, far from being selectively activated by noxious heat (45–50 °C), PEP neurons typically detected the full temperature range (Fig. 1c–e), exhibiting graded responses that increased in magnitude and duration across both the innocuous (37–42 °C) and noxious ranges (Fig. 1c,d). PEP constituted only a small subset of the heat-responsive cells. For example, just as many itch-related^10,24^ cells expressing *Nppb* and *Sst* (NP3) exhibited responses across the full temperature range (Fig. 1c–e). Moreover, half the noxious heat-responsive cells were in three other non-peptidergic classes (*Mrgprd*-, *Mrgpra3*- and *Mrgprb4*-expressing cells (NP1, NP2A and NP2B, respectively)), which are not generally considered to be thermonociceptors^4,5,21,25^ despite reports suggesting thermosensitivity^22,26^ (Fig. 1c–e). In fact, in the noxious temperature range, heat responses were dominated by classes other than the PEP nociceptors (Fig. 1e).

## Non-canonical thermosensors

NP1 is a transcriptomically homogeneous class of mouse somatosensory neurons that has been suggested to function primarily in mechanonociception^21^ and to have a minor role in thermonociception^22,27^. NP2B neurons are transcriptomically similar to NP2A itch-related cells^11^ but have been reported to detect brush but not pinch^4^ and to have a role in affective touch and sexual behaviour^5^. By contrast, our data (Fig. 1) indicate that NP1 and NP2B neurons innervating the cheek have closely related activity profiles, preferentially responding to high-threshold mechanical stimuli and noxious heat. A recent study also suggested that NP1- and NP2B-lineage neurons have similar receptive tuning^28^. To better understand these differences and to benchmark our ISH-based approach, we crossed the two Cre lines^4,29^ that were used in previous studies^4,5,28^ into the Ai95 (RCL-GCaMP6f) background^30^.

**Fig. 2:**
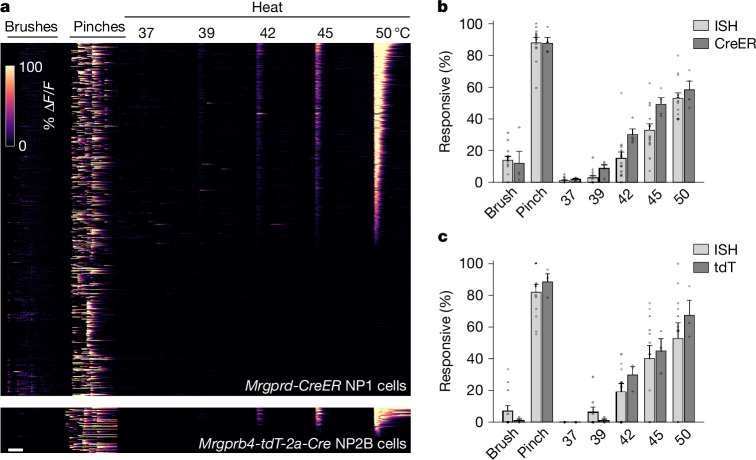
In vivo functional imaging of Cre lines confirmed that NP1 and NP2B neurons detect noxious mechanical and thermal stimuli. **a**, Heat maps showing calcium transients from Ai95 carrying knock-in alleles to label NP1 (upper panel; 561 neurons; four mice) and NP2B (lower panel; 72 neurons; three mice) neurons. Changes in GCaMP fluorescence in response to stimuli applied to the cheek are colour-coded by % Δ*F*/*F* (inset scale). **b**,**c**, Percentage of neurons (mean ± s.e.m.) responding to each stimulus for cells identified by ISH after functional imaging (pale grey) or Cre-mediated labelling (dark grey). **b**, NP1 cells: ISH, *n* = 13; CreER, *n* = 4. **c**, NP2B cells: ISH, *n* = 12; tdT, *n* = 3. See Supplementary Table 2 for details and statistics. Scale bar, 10 s.

**Extended Data Fig. 3:**
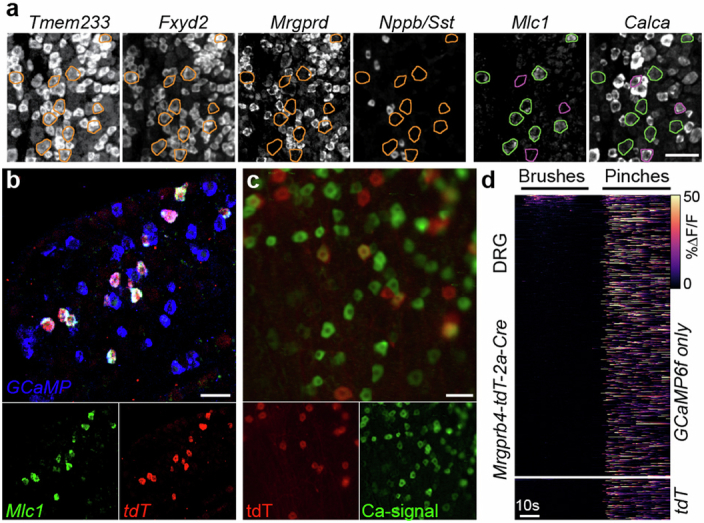
NP2B neurons, specificity of *Mrgprb4-tdT-Cre* recombination and DRG responses. (a) Whole mount ISH images comparing methods used to distinguish NP2A and NP2B cells. NP2 cells (orange outlines) can be assigned by expression of *Tmem233* and *Fxyd2* but not *Mrgprd* or *Nppb/Sst*. NP2B cells (green outlines) also express *Mlc1* and a lower level of *Calca* than NP2A cells (magenta outlines); scale bar = 50 µm. In animals where both methods were applied, there was >90 % agreement. (b) Example triple label ISH of a section from an Ai95 mouse carrying an *Mrdprb4-tdT-Cre* allele showing *GCaMP* (blue), *Mlc1* (green) and *tdT* (red). Note perfect overlap of *Mlc1* and *tdT* showing that *tdT* marks NP2B cells; *GCaMP* is much more broadly expressed. (c) Example image showing alignment of in vivo tdT fluorescence (red) and a spatial map of stimulus evoked activity (green, all stimuli combined) showing that most responding neurons in these mice are not NP2B cells; scale bars (b, b) = 50 µm for merged views. (d) Heatmap showing Ca-transients from lumbar DRG neurons from Ai95 mice (n = 5) carrying the *Mrdprb4-tdT-Cre* allele divided into 747 GCaMP-only cells and 109 NP2B neurons expressing tdT; changes in GCaMP fluorescence (% ΔF/F) are color-coded as indicated in the scale bar.

**Extended Data Fig. 4:**
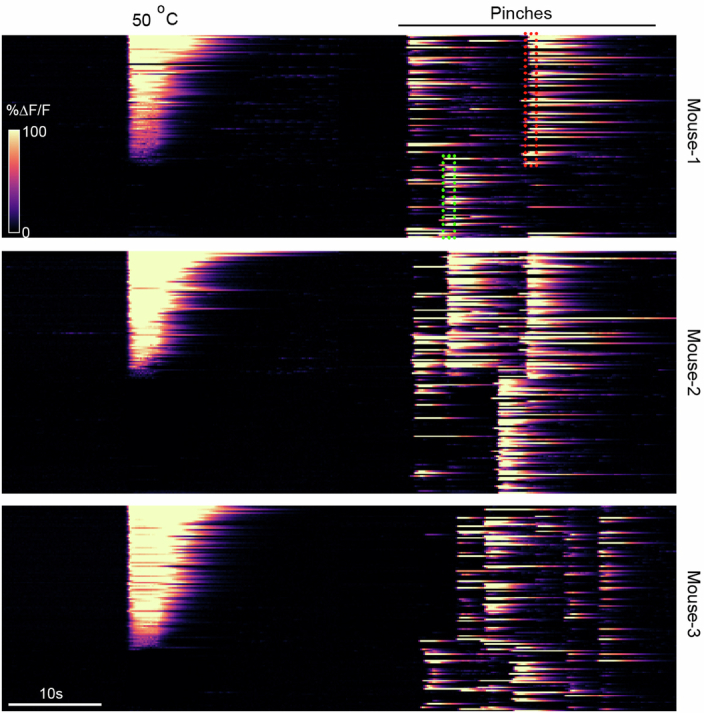
Tuning of NP1 neurons reflects stimulus application rather than heterogeneity in this class of sensory neurons. Heatmaps showing Ca-transients from trigeminal neurons of three Ai95 mice carrying the *Mrgprd-CreER* knockin allele after tamoxifen induction; changes in GCaMP fluorescence in response to stimuli applied to the cheek are color-coded by % ΔF/F. Responding neurons were sorted based on their response to heating. Note that for each mouse, there were individual pinches that primarily activated neurons that were not temperature responsive (an example boxed, green) and other pinches that generally activated temperature responsive neurons (an example boxed, red). This strongly suggests that all NP1 neurons innervating the cheek are polymodal and that differences in position of stimulus activation or size of thermal versus mechanical receptive field account for apparent variation in tuning selectivity.

The well-characterized *Mrgprd-CreER* line faithfully captures NP1 neurons in adult mice^28,29^. Functional recordings from these cells (Fig. 2a,b) were consistent with the results of the ISH-based approach (Fig. 1), confirming that NP1 cells respond to noxious thermal and mechanical stimulation. The *Mrdprb4-*tdT*-2a-Cre* line that was used to target NP2B cells^4,5,28^ mediates Cre recombination more broadly (Extended Data Fig. 4). However, although tdTomato (tdT)-positive neurons were a small subset of GCaMP-expressing cells, 99% of tdT cells (396 of 400 neurons from three mice) expressed *Mlc1*, a marker of NP2B cells (Extended Data Fig. 3a,b). Functional imaging of tdT-positive neurons supported the results from the comprehensive analysis of neuronal function (Fig. 1) by showing that NP2B neurons were not selectively activated by gentle stimulation of the cheek but instead responded most strongly to higher-intensity mechanical stimuli and heating (Fig. 2a). Quantification of responses (Fig. 2c) confirmed close correspondence between ISH and Cre recombination-based datasets. Thus, NP1 and NP2B neurons detect noxious stimuli applied to the hairy skin of the cheek.

Previous studies of NP2B neurons^4,5,28^ investigated peripheral targets of dorsal root ganglia (DRG) rather than the trigeminal ganglion, raising the possibility that DRG and trigeminal NP2B neurons are differentially responsive to mechanical stimuli. Therefore, we recorded lumbar DRG responses of *Mrdprb4*-tdT-*2a*-*Cre* labelled neurons to mechanical stimulation of hairy skin (Extended Data Fig. 3d). Responses from the full lineage were consistent with recently published results^28^ showing a small fraction (approximately 5%) to be brush-sensitive. However, when we analysed NP2B neurons (tdT-positive cells), they exhibited pinch responses but were unresponsive to brushing (Extended Data Fig. 3d), similar to their trigeminal counterparts, further demonstrating that these neurons are not selective gentle touch receptors^4^.

NP1 neurons are divided into polymodal and pinch-selective functional types, despite their homogeneous transcriptomic profile^10–12^ (Figs. 1b and 2a). We used a series of pinches to provide a naturalistic mechanical stimulus to a broad area of the cheek and applied the thermal probe to a similar area of the skin. However, differences in stimulus location, rather than biological differences, may contribute to the impression that some cells are narrowly tuned. We observed individual pinches that almost exclusively activated polymodal cells and other pinches (at different locations) that selectively recruited heat-insensitive nociceptors (Extended Data Fig. 4). This suggests that differential tuning of NP1 neurons can largely be explained by their small receptive field^3,29^ coupled with our naturalistic stimulation approach (Methods). Other classes of C-nociceptors also included both selective and polymodal cells but with quite different proportions tuned to heat or pinch (Fig. 1b). We suggest that the long-standing debate about the nature of polymodal and narrowly responsive nociceptors^31,32^ simply reflects the mode of stimulus application coupled with the relative sensitivity of different classes of nociceptor to thermal versus mechanical stimuli and resultant differences in their effective receptive fields for these two modalities.

Taken together, detailed analysis of how the different types of C-nociceptors respond to heat and noxious mechanical stimulation (Figs. 1 and 2) challenges the view that various transcriptomic classes have distinct and independent roles in thermo- and mechanosensory detection and discrimination^4,5,22,24,25,33^. Instead, our data suggest that all classes of C-nociceptors are broadly tuned with overlapping but distinct response profiles much like the cones in the human visual system^34^. By analogy, such tuning should allow efficient and reliable encoding of multiple stimulus features, including modality, position, extent and intensity, which are important for triggering appropriate behavioural responses. Moreover, noxious mechanical stimuli, but not thermal stimuli, recruit an array of low-threshold mechanoreceptors (LTMRs), augmenting the discriminatory power of the system.

## Nociceptor activity during inflammation

A crucial role of the somatosensory system is to evoke pain as a protective mechanism for preventing tissue damage and promoting healing^2,35^. For example, injury and inflammation result in continuing localized pain, alter the evaluation of sensory input and evoke a range of attending behaviours^35^. It is well known that sensory afferents dramatically change their firing properties under these conditions^2,32,36^. To better characterize these changes, we carried out longitudinal imaging to track and classify which neurons are affected by injection of the fast-acting inflammatory mediator prostaglandin E2 (PGE2). This paradigm induces swelling, spontaneous pain behaviour, hyperalgesia and allodynia within 10 min of injection with recovery after about 2 h (ref. ^37^).

**Fig. 3:**
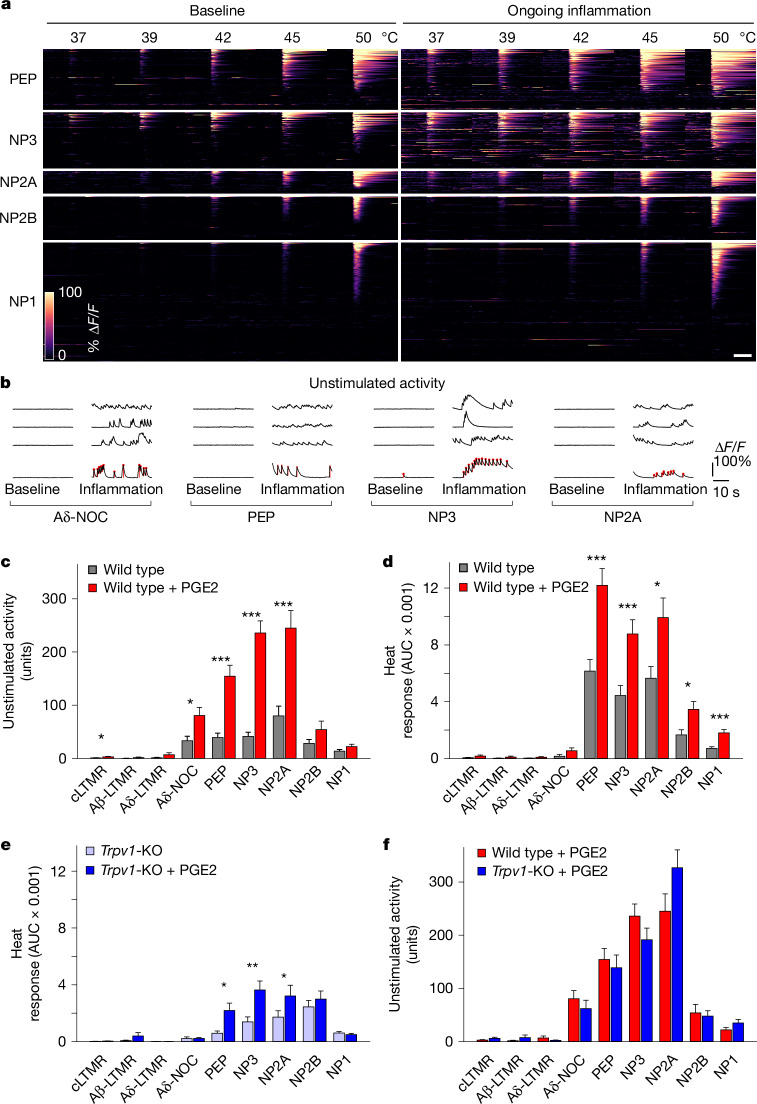
PGE2-mediated inflammation differentially affects select classes of nociceptors. **a**, Heat maps showing the effect of PGE2-induced inflammation on the thermal sensitivity of 582 C-nociceptors (eight mice) that responded to heat and/or pinch of the cheek (cheek-innervating neurons) grouped by class; for display purposes, baseline (left) and continuing inflammation (right) were independently sorted on the basis of the amplitude of heat response. **b**, Example of GCaMP traces for individual neurons at baseline (left) and after PGE2 injection (right) for classes with spontaneous activity during inflammation; red points and bars in the lowest example trace identify transients and their amplitudes (see Methods for details). **c**, Quantification of unstimulated activity (mean sum of transient amplitudes ± s.e.m.) in neuronal classes in wild-type mice before (grey) and after (red) PGE2-induced inflammation. **d**,**e**, Heat response for all cheek-innervating neurons of a given class over the full temperature range (mean area under the curve (AUC) ± s.e.m.). Wild-type mice (**d**) and *Trpv1*^−/−^ mice (**e**) before and after PGE2 injection of the cheek. Additional information about heat sensisitization is shown in Extended Data Figs. 5 and 6. **f**, Unstimulated activity (mean sum of transient amplitudes ± s.e.m.) in neuronal class activity during inflammation in wild-type mice (red) and *Trpv1*^−/−^ mice (blue). Note that the red bars in **c** and **f** show the same dataset for comparative purposes; *P < 0.05; **P < 0.01; ***P < 0.001; for details of statistical tests and number of mice and cells, see Supplementary Tables 1 and 2. KO, knockout. Scale bars, 10 s.

Most sensory neurons that respond to stimulation of the cheek were quiescent when held at 30 °C under baseline conditions. By contrast, after PGE2 injection, many cheek-innervating nociceptors became active in the absence of applied stimuli (Fig. 3a,b and Supplementary Video 1). Spontaneous activity developed over the first 5 min and lasted for more than an hour. To quantify the magnitude of spontaneous activity, we assessed both the frequency and amplitude of responses (Fig. 3b). This analysis (Fig. 3c) revealed that spontaneous activity was almost entirely restricted to nociceptors (primarily Aδ-NOC, PEP, NP2A and NP3) but not LTMRs, providing a compelling explanation for localized continuing pain associated with inflammation. PGE2 receptor expression^11–13^ does not match the classes of neurons displaying spontaneous activity, suggesting that indirect inflammatory effectors are probably involved.

**Extended Data Fig. 5:**
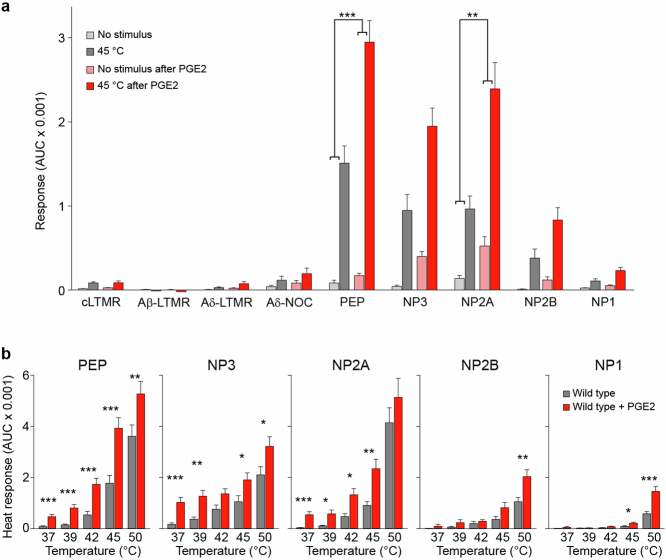
Inflammatory heat sensitization and sensory stimulus ongoing activity of nociceptors in wild type mice. a) Response magnitude (mean AUC ± s.e.m) at the holding temperature of 30 °C (No stimulus) and at 45 °C before (light or dark grey) and after PGE2 injection (pink or red) expose the relative contributions of ongoing activity and thermal sensitization that occur in this model of inflammatory pain. There was a significant increase in the magnitude of the temperature response (response at 45 °C minus response without stimulation) in PEP and NP2A nociceptors after PGE2 induced inflammation. (b) Heat response magnitude (mean AUC ± s.e.m) for wild type mice before (grey) and after (red) PGE2 injection to the cheek. p < 0.05, *; p < 0.01, **; p < 0.001, ***; for details of statistical tests and numbers of mice and cells see Supplementary Information, Tables 1 and 2.

PGE2 injection potentiated heat detection by C-nociceptors (Fig. 3a,d), which is consistent with previous results^31^. This can be clearly observed in the time-locked temperature responses and is superimposed on the generally lower-magnitude spontaneous nociceptor activity induced by PGE2 (Fig. 3a and Extended Data Fig. 5a). All other classes of neurons remained insensitive to heat (Fig. 3d). A more detailed analysis (Extended Data Fig. 5b) revealed that responses to temperatures in the normally innocuous, warm range were increased in PEP, NP2A and NP3, suggesting a cellular logic for inflammatory thermal allodynia. PGE2 injection also causes heat hyperalgesia at noxious temperatures (45–50 °C) characterized by a variety of exaggerated responses and coping behaviours^2^. Over this temperature range, our results indicate that NP1 and NP2B also contribute to elevated pain responses (Extended Data Fig. 5b).

**Extended Data Fig. 6:**
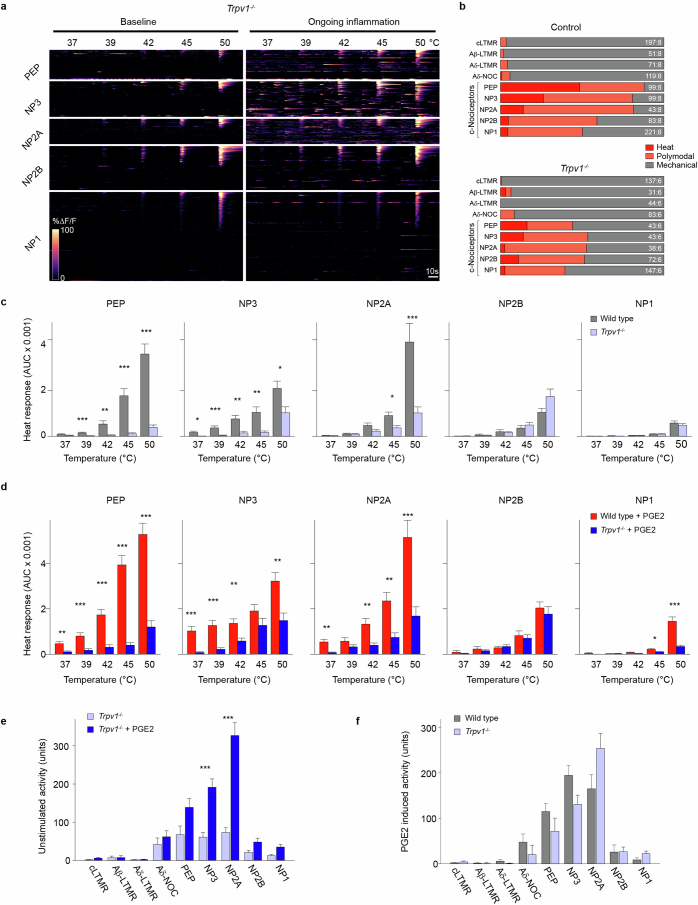
Inflammatory heat sensitization and sensory stimulus ongoing activity of nociceptors in *Trpv1*^-/-^ mice. a) Heatmaps showing the effect of PGE2-induced inflammation on the thermal sensitivity of 371 C-nociceptors in 6 *Trpv1*^*-/-*^ mice that responded to heat and/or pinch of the cheek grouped by class; for display purposes baseline (left) and ongoing inflammation (right) were independently sorted based on magnitude of heat response. (b) Proportions of heat (red), polymodal (orange) and mechanically tuned neurons (grey) for each class in wild type mice (upper panel) or *Trpv1*^*-/-*^ mice (lower panel); the number of neurons and mice used for assignment are indicated. Note that the relative number of PEP and NP3 neurons as well as the proportion responding to temperature is reduced in the knockout animals. (c, d) Heat response magnitudes (mean AUC ± s.e.m) displayed to compare wild type and *Trpv1*^*-/-*^ mice (c) before and (d) after PGE2 injection to the cheek; note that the wild type data are a repeat of the data displayed in (Extended Data Fig. 6b). (e) Quantitation of unstimulated activity in *Trpv1*^*-/-*^ mice (mean sum of transient amplitudes ± s.e.m.) before (pale blue) and after PGE2 induced inflammation (dark blue). (f) Change in unstimulated activity induced by PGE injection of the cheek (mean sum of transient amplitudes ± s.e.m.) for wild type (grey) and *Trpv1*^*-/-*^ (blue) mice; p < 0.05, *; p < 0.01, **; p < 0.001, ***; for details of statistical tests and numbers of mice and cells see Supplementary Information, Tables 1 and 2.

PEP, NP2A and NP3 neurons express the ion channel TRPV1, which has widely been reported to have only a minor role in baseline thermal behaviour^22,38,39^ but is essential for heightened responses to heat after inflammation^38,39^. Therefore, we next tested how the peripheral representation of heat and inflammatory sensitization was affected by the absence of this ion channel. Trigeminal neuron responses to heat were dramatically reduced in *Trpv1*^−/−^ mice (Fig. 3e), with smaller proportions of PEP, NP3 and NP2A responsive to temperature and changes in the relative tuning of these neuron classes to heat and pinch (Extended Data Fig. 6a–c). These large-scale changes were surprising given the modest effects of *Trpv1* knockout on thermal behaviour^22,38,39^ and suggest that the small amount of residual nociceptor activity (Fig. 3e and Extended Data Fig. 6), perhaps combined with the inhibition of cooling-responsive neurons^40^, provides sufficient heat discrimination for many behavioural paradigms. Notably, PGE2-induced inflammation had only modest effects on the temperature sensitivity of nociceptors in *Trpv1*^−/−^ animals (Fig. 3e), which explains the crucial role of this ion channel in inflammation-related thermal allodynia and hyperalgesia^38,39^. This was also true for the sensitization of NP1 and NP2B neurons (Fig. 3e), which did not prominently express *Trpv1*. We suggest that low levels of this ion channel in these cell types or perhaps non-cell autonomous effects, for example, neuroinflammation^41^, contribute to heat hyperalgesia. Finally, unlike for heat sensitization, where TRPV1 has a major role, the stimulation of continuing nociceptor activity after PGE2 injection was largely unaltered in *Trpv1*^−/−^ animals (Fig. 3f and Extended Data Fig. 6e,f). Thus, PGE2 must affect more than one signalling pathway in nociceptors, including one that generates spontaneous activity and another that potentiates heat responses through TRPV1.

## Logic for inflammatory tactile allodynia

**Fig. 4:**
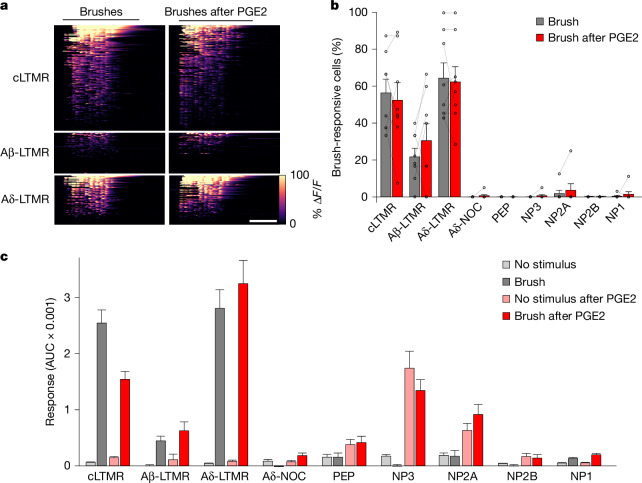
Stable representation of gentle touch at the periphery during inflammation. **a**, Heat maps showing the effect of PGE2-induced inflammation on the brush sensitivity of 339 LTMRs (eight mice) that responded to pinch and/or brush of the cheek grouped by class. **b**, Percentage of neurons responding to at least 50% of individual brushes before (grey) and after (red) PGE2; mean ± s.e.m.; *n* = 8. **c**, Brush response magnitude (mean AUC ± s.e.m.) for all cheek-innervating neurons of a given class before (grey) and after (red) PGE2 injection; background and PGE2-induced activity (mean AUC ± s.e.m.) during an equivalent period without stimulation (pale grey and pink) showed no increase in LTMR responses after PGE2 injection and that nociceptor firing during inflammation is largely brush-independent (Extended Data Fig. 7). Supplementary Tables 1 and 2 include full details of statistical tests and numbers. Scale bar, 10 s.

Tactile allodynia, a major consequence of many types of inflammation^2^, is dependent on signalling through the mechanosensory ion channel PIEZO2 (refs. ^42,43^). Therefore, we evaluated whether the detection of gentle brushing, a stimulus that elicits pain-related behavioural responses during inflammation^2^, was affected by PGE2 injection. Qualitatively, there were minimal changes in LTMR calcium transients (Fig. 4a), although quantification revealed a slight decrease in C-LTMR responses but no significant changes in Aδ- and Aβ-LTMR activity (Supplementary Table 2). Thus, although it is possible that small differences in the firing of Aβ-neurons^44^ contribute to inflammatory mechanical allodynia, such changes are no larger than natural variation in brush responses of LTMRs (Fig. 4a).

**Extended Data Fig. 7:**
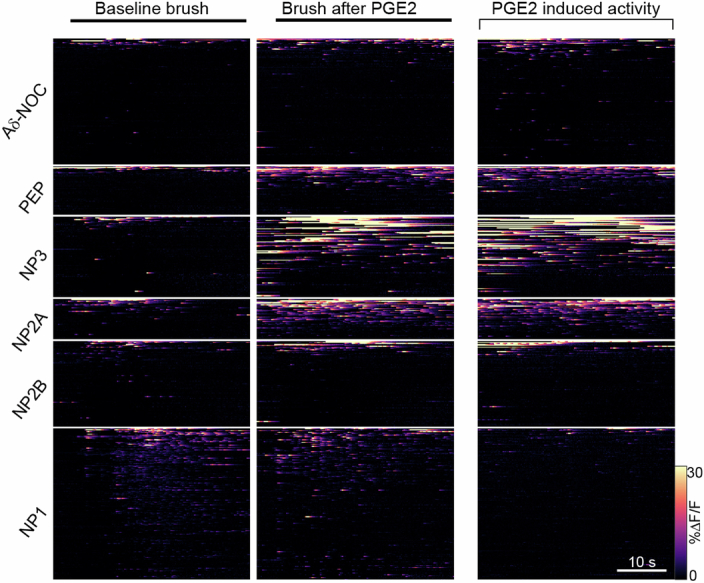
Nociceptor responses to brushing are not increased during PGE2 induced inflammation. Heatmaps showing the effect of PGE2-induced inflammation on the brush sensitivity and stimulus independent activity (right column) of 580 nociceptors (from 8 mice) that responded to mechanical stimulation of the cheek grouped by class. Note that spontaneous activity in Aδ-NOC, PEP, NP3, NP2A and NP2B neurons accounts for apparent activity during brushing i.e., brush responses were not increased during inflammation. Weak brush activity in NP1 neurons was not changed after PGE2 injection (see Fig. 4c).

We next analysed whether nociceptors are sensitized to brush after PGE2. Here, inflammation-related continuing activity in nociceptors complicates the analysis. However, unlike LTMRs, very few nociceptors reliably detected brushing, that is, responded during 50% or more brushes, either at baseline or after PGE2 (Fig. 4b). Moreover, comparison of response profiles (Extended Data Fig. 7) and quantification of calcium transients (Fig. 4c) demonstrated that brushing after PGE2 injection did not augment the continuing activity or mechanical sensitivity of any nociceptor class (see Supplementary Table 2 for statistical analysis). Because gentle touch remains largely unchanged during inflammation, the question remains how PGE2 injection changes the behavioural response of mice to this type of stimulation^37^. Notably, our data (Fig. 4c and Extended Data Fig. 7) showed that inflammation-induced continuing activity alters the population of peripheral neurons that are active during brushing. Therefore, we reasoned that this touch-independent nociceptor activity may transform the central (for example, spinal cord processing) of normal LTMR input to serve as a driver of localized pain. This new model is completely consistent with the crucial role of PIEZO2 (refs. ^42,43^) and the importance of LTMRs^44^ in inflammatory tactile allodynia.

**Fig. 5:**
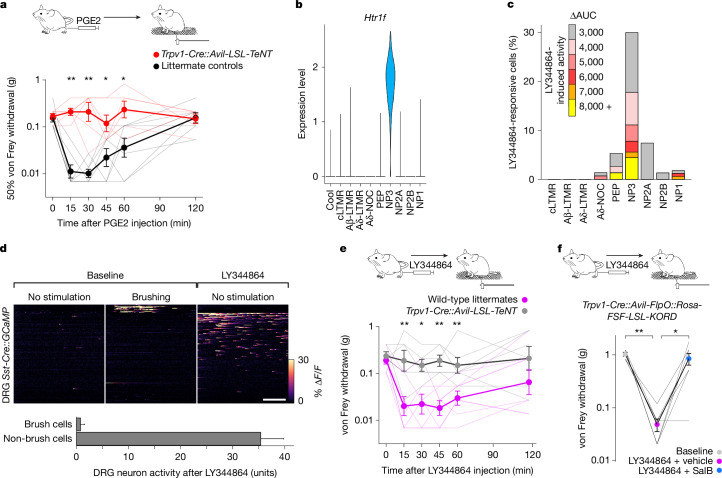
Continuing nociceptor activity drives tactile allodynia. **a**, Time course of von Frey 50% withdrawal threshold (mean ± s.e.m.) before (time = 0) and after injection of PGE2 into the hind paw of mice with silenced *Trpv1*-lineage nociceptors (red; *n* = 6) and littermate controls (black; *n* = 7). PGE2 injection induced mechanical sensitization in controls but not in mice with silenced nociceptors (*P* < 0.05) over 60 min. **b**, Violin-plot analysis of expression level (log-normalized single-cell RNA sequencing data)^11,28^ showing selective expression of *Htr1f* in NP3 nociceptors. **c**, Percentage and level of activation (ΔAUC) of neurons after injection of the selective HTR1F agonist LY344864 into the cheek. **d**, Heat maps of DRG imaging for mice expressing GCaMP under the control of *Sst*-*Cre*. Responses from 200 neurons of six mice showed that *Sst*-*Cre* labelled a population of brush cells and a separate population of LY344864-responsive neurons. Below, quantification of LY344864-stimulated activity (mean sum of transient amplitudes ± s.e.m.) for brush and non-brush cells. **e**, von Frey 50% withdrawal threshold (mean ± s.e.m.) before (time = 0) and after injection of LY344864 into the hind paw of wild-type mice (magenta; *n* = 7) and littermates with silenced *Trpv1*-lineage nociceptors (grey; *n* = 6). LY344864 injection induced mechanical sensitization in wild-type mice for at least 60 min but was ineffective in mice with silenced nociceptors. **f**, von Frey 60% withdrawal thresholds (mean ± s.e.m.; *n* = 7) at baseline (grey) and after paw injection of LY344864 in mice expressing KORD^53^ in *Trpv1*-lineage nociceptors. Mice received intraperitoneal dimethylsulfoxide (vehicle; magenta) or the selective KORD ligand Salvinorin B (SalB; blue) immediately before paw injection of LY344864. See Supplementary Tables 1 and 2 for full details of statistical tests and numbers; *P < 0.05; **P < 0.01. Scale bar, 10 s.

**Extended Data Fig. 8:**
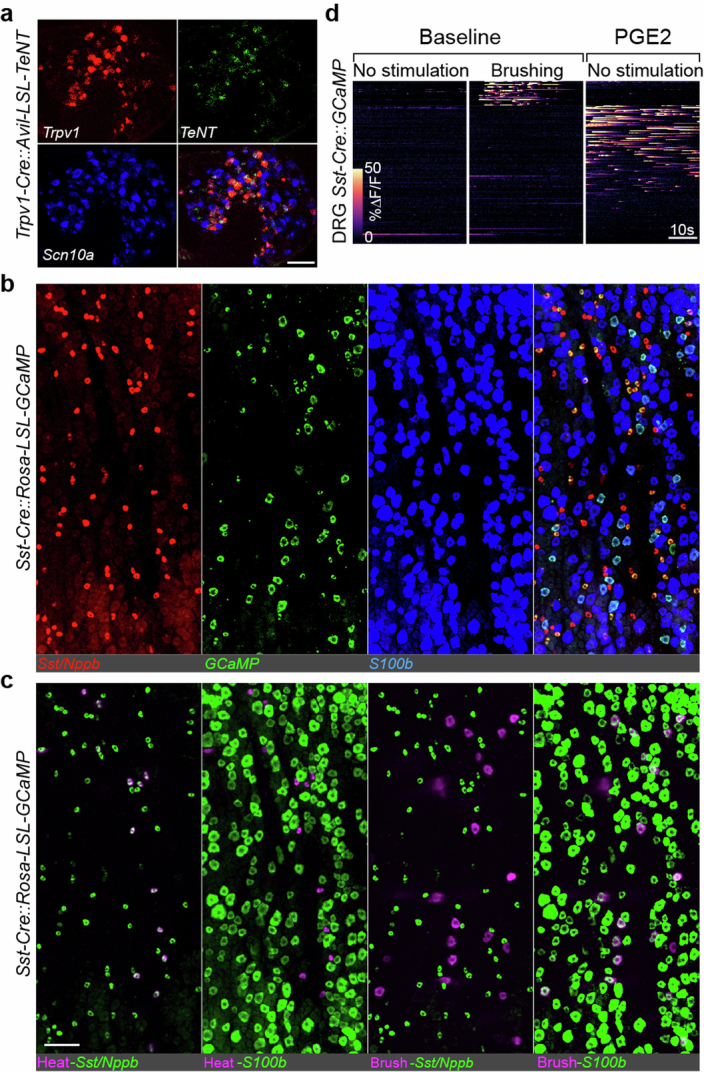
Silencing nociceptors and targeting NP3 cells as controls for strategies used to functionally determine nociceptor contribution to allodynia. (a) Triple label ISH validating our approach for silencing a large subset of nociceptors (*Trpv1* expressing PEP, NP3 and NP2A neurons). In DRGs, >96% of *Trpv1* expressing neurons were *TeNT* positive (624/643 cells in sections from 3 mice) and >97% of *TeNT* positive cells were nociceptors i.e., co-expressed *Trpv1* and/or *Scn10a*. (b) *Sst-Cre* was used to target NP3 cells; Cre-recombination (green) occurred in 63% of NP3 neurons expressing *Sst/Nppb* (red, 246/389) with variable (50-80%) recombination across 3 animals. Moreover, NP3 cells accounted for only 29% of the recombined neurons (246/847). Recombination also labeled *S100b*-positive neurons (blue), which do not express *Sst*, these cells were large-diameter and accounted for the other 71% of recombined cells. (c) Functional responses (magenta) were aligned to gene expression (green) showing that small diameter *Sst/Nppb*-NP3 cells never respond to brush but are heat sensitive consistent with data in Fig. 1. Brush but not heat activates the large diameter *S100b*-expressing neurons; scale bars, (a, c) 50 µm. (d) Heatmaps of DRG imaging for mice expressing GCaMP under the control of *Sst-Cre*. Responses from 174 neurons from 7 mice show that *Sst-Cre* labeled neurons are spontaneously active after injection of PGE2 into the paw, but brush cells were not activated by inflammation.

**Extended Data Fig. 9:**
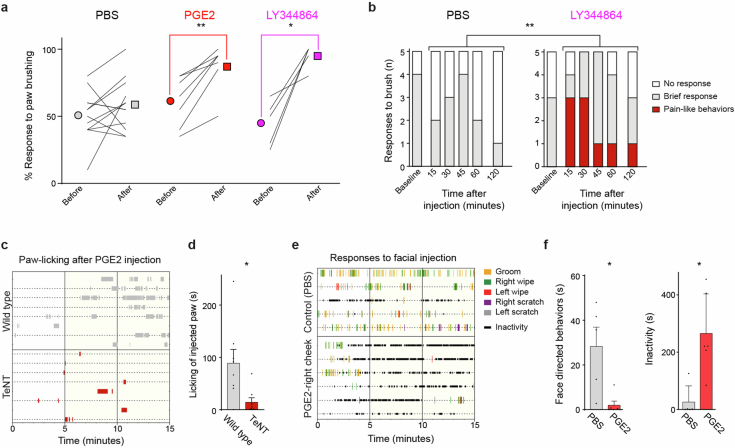
Dynamic tactile allodynia induced by PGE2 or LY344864 and unstimulated behavioral responses to inflammation. a) The right hind paws of C57B/6 mice were brushed 20 times before and after injection of PBS (vehicle, n = 12), PGE2 (n = 7) or LY344864 (n = 5). Percentage response of each mouse (black lines) and the mean response of the group before (circles) and after injection (squares) are shown. (b) Time course showing that hind paw injection of LY344864 but not PBS induces pain-like behaviors to gentle brush (repetitive or extended lifting and guarding) resembling the time course for von Frey sensitization (Fig. 5e). (c-f) Changes in behavior were observed following (c,d) hind paw and (e, f) facial injection of PGE2 in wild type mice. (c) Ethograms showing time spent licking the injected hind paw for wild type mice (grey bars, n = 8) and littermates expressing TeNT in nociceptors (red bars, n = 8) for 15 min after injection of PGE2. Note that licking of the injected hind paw develops slowly minutes after PGE injection corresponding to induction of inflammation^37^ and is rarely seen in TeNT mice where nociceptor signaling is blocked. (d) Quantification of time spent licking the affected paw (mean ± s.e.m.) for the 10-minute period at the start of inflammation highlighted in yellow in panel (c). (e) Ethograms showing scored face directed behaviors, grooming of the face with both front paws (yellow), wiping with the right (green) or left (red) front paw and hind paw scratching of the right (purple) or left cheek (grey) for 15 min after facial injection of PBS (n = 5) or PGE2 (n = 6). Also shown are periods of inactivity greater than 1 s (black). Note that mice injected with PGE2 became inactive, often standing in a hunched posture within 5 min of PGE2 injection (Supplementary Information, Videos 2, 3), mirroring the induction of inflammation^37^ and nociceptor activity (Fig. 3a). Notably, PGE2 induced inflammation did not elicit the wiping or scratching observed after injection of strong agonists of select nociceptor classes^46^ but instead significantly reduced face directed behaviors. Anecdotally, PGE2 injected animals were sometimes observed to raise their paw towards the injected cheek but refrain from touching the skin (Supplementary Information, Video 4). (f) Quantitation of time spent in face directed behavior (left) and inactivity (right) after PBS (grey) or PGE2 (red) for the 10-minute period highlighted in yellow in panel (e). p < 0.05, *; p < 0.01, **; for details of statistical tests and numbers of mice see Supplementary Information, Tables 1 and 2.

The hypothesis that coincident firing of LTMRs with nociceptors is the basis for tactile allodynia during inflammation makes two testable predictions. First, if spontaneous nociceptor firing drives tactile allodynia, silencing these neurons should block sensitization. Therefore, we developed an intersectional approach to express a fragment of the tetanus toxin (TeNT) that potently blocks synaptic transmission in nociceptors (Extended Data Fig. 8a). These mice have no gross motor deficits and exhibit normal baseline von Frey responses (Fig. 5a). Just as predicted, PGE2 injection to the hind paw failed to trigger tactile allodynia in these TeNT-expressing mice (Fig. 5a), whereas littermate controls exhibited strong sensitization. Second, we reasoned that any continuing activity in nociceptors should be sufficient to trigger touch-evoked pain. Here we took advantage of the unique molecular expression profile of NP3 neurons, one of the cell classes most strongly activated by PGE2 injection (Fig. 3e). It was recently reported that LY344864, a synthetic agonist for the serotonin receptor HTR1F, which is specifically expressed in NP3 cells (Fig. 5b), triggers itch through activation of this cell class^45^. We demonstrated that local subcutaneous injection of LY344864 in the cheek potently activated trigeminal NP3 neurons with high selectivity (Fig. 5c). Using an *Sst*-*Cre* driver to target these cells in the DRG, we showed that both LY344864 and PGE2 induced NP3 firing with comparable intensity and duration (Fig. 5d and Extended Data Fig. 8b–d). Notably, LY344864 also induced potent tactile allodynia when injected into the hind paw (Fig. 5e and Extended Data Fig. 9a,b). Moreover, blocking synaptic transmission in these cells (Fig. 5e) or acute inhibition of these cells in adult mice (Fig. 5f) abolished the LY344864-mediated sensitization, confirming the importance of NP3 nociceptors in mediating inflammatory allodynia in this model. Taken together, these results show how touch-independent nociceptor activity can make a normally innocuous mechanical stimulus painful, without changing the way this stimulus is detected at the periphery.

Finally, we explored whether spontaneous nociceptor firing drives inflammatory pain in the absence of externally applied stimulation. As predicted, hind paw injection of PGE2 evoked typical pain behaviours, including copious paw licking in wild-type mice but not in TeNT littermates in which nociceptors were silenced (Extended Data Fig. 9c,d). When injected in the cheek, PGE2 did not immediately evoke the wiping response typically associated with painful agents^46^. Instead, within 5 min of injection, face-directed behaviours (wiping, grooming and scratching) were almost completely suppressed (Extended Data Fig. 9e,f), with mice often standing motionless and hunched for extended periods (Supplementary Videos 2–4). Taken together, these results support a role for spontaneous nociceptor firing as a driver for continuing inflammatory pain.

## Discussion

One of the most remarkable features of the somatosensory system is its ability to encode and discriminate diverse physical stimuli while also providing information about the position, extent, pleasant versus aversive nature and intensity of the stimulus^1,2,8,35^. Here we combined functional imaging with transcriptomic classification to dissect the cellular logic by which touch and heat are encoded at the periphery and how this representation is altered during inflammation.

Simultaneously interrogating the response properties of all the major types of cutaneous somatosensory neurons to complex stimuli revealed key features underlying the coding of heat and noxious mechanical stimuli. First, our data show the complete segregation of cell classes that respond to gentle mechanical stimulation from those that detect heat. Second, although *Trpv1*-expressing neurons are particularly sensitive to temperature and reliably detect warming, several other types of C-nociceptors are prominently activated by noxious heat. Interestingly, *Mrgprb4*-expressing NP2B cells that have previously been linked to gentle stroking, affective touch and sexual response^4,5^ have the receptive tuning of nociceptors rather than LTMRs throughout the body. Third, matching receptive tuning to cell class allows differential effects of functionally important molecules in distinct classes of cell to be determined^3^. Using knockout mice, we showed that the capsaicin receptor TRPV1 is largely responsible for the particular thermosensitivity of PEP, NP2A and NP3 neurons. In keeping with its function in the oral cavity^47^, TRPV1 mediates almost all responses to innocuous warming but, in contrast to predictions from its in vitro threshold^7^, has a lesser role in the detection of noxious heat. Together, these data showed that, rather than classes of nociceptor being selectively tuned to a single modality^4,5,24,25^, there was an inverse relationship between mechanical and thermal sensitivity, such that all types of C-nociceptors responded to heat and noxious mechanical stimulation, albeit with differential graded tuning. The distinct but overlapping response spectra of cell classes imply that the somatosensory system uses combinatorial coding to achieve its remarkable discriminatory power.

Changes in the firing patterns of somatosensory neurons have long been linked to peripheral inflammation and are assumed to drive associated pain^2,8,32,36^. Our analysis demonstrated that PGE2-mediated inflammation induces stimulus-independent activity in several types of nociceptors and sensitizes these cells to heat but not gentle mechanical stimulation. Inflammatory heat sensitization was greatly reduced in *Trpv1*^−/−^ mice (Fig. 3d,e), matching behavioural measures of heat allodynia and hyperalgesia during inflammation^38,39,48^. By contrast, spontaneous activity, which we considered as important for continuing inflammatory pain, was well preserved in the knockout mice (Fig. 3c,f). Thus, TRPV1 antagonists might be expected to alleviate heat sensitization but be less effective for other aspects of inflammatory pain. Our results showed that spontaneous nociceptor firing drives inflammatory tactile allodynia, and eliminating nociceptor input blunts this sensory transformation (Figs. 4 and 5). These findings are consistent with the crucial role of PIEZO2 in this type of pain^42^, despite its relatively minor role in mechanonociception^3,17,43^. It also helps to explain how excitotoxic-mediated ablation of nociceptors can be effective in treating several forms of pain without substantially affecting gentle touch or mechanonociception^49^.

There are limitations to our study. For example, we only studied the functional coding of neurons innervating hairy skin using functional imaging. Calcium transients provide a reliable proxy for neuronal firing^3,19^ but may miss subtle effects that could contribute to pain^44^. However, in day-to-day life, subtle variations in LTMR firing provide discriminatory information without normally causing pain, further supporting our hypothesis. We also note that although our dissection of cell classes was as comprehensive as that in a recent study using Cre drivers^28^, further subdividing the Aβ-LTMRs, Aδ-NOC and PEP populations may reveal more nuances. In the future, extending these findings to glabrous skin and internal targets of somatosensory neurons (bone, muscle and viscera) will also be important. Similarly, we examined a short-term inflammatory model of pain, but there are many longer-term disease states that cause pain in humans, including diabetes, cancer and blood disorders (for example, sickle cell disease). Dissecting the extent to which these pathologies induce similar changes in sensory coding across cell classes should be informative for developing appropriate pain therapies. It will also be interesting to determine how inputs from the various heat-responsive nociceptors converge in the dorsal horn and spinal trigeminal nucleus to support combinatorial coding and how inflammation-dependent spontaneous firing might alter this representation. Finally, all these studies were performed in mice; human somatosensory neurons exhibit differences at the molecular^50^ and class-based^51,52^ levels, which may affect the precise details. However, the underlying principles we report, including distributed coding of heat across nociceptor classes and selective effects of inflammation on nociceptor activity and thermal sensitivity, will likely be relevant for human sensation and pain.

## Methods

### Experimental animals

Experiments using animals were performed in accordance with the guidelines set forth by the National Institutes of Health and approved by the National Institute of Neurological Disorders and Stroke or the National Institute of Dental and Craniofacial Research Animal Care and Use Committees. Mouse lines Ai95(RCL-GCaMP6f)-D (no. 024105)^30^, *Mrgprd*-*CreERT2* (no. 031286)^29^, *Mrdprb4*-tdT-*2a*-*Cre* (no. 021077)^4^, *Trpv1*-*Cre* (no. 017769)^54^, *Sst*-*Cre* (no. 013044)^55^ and *Trpv1*^−/−^ (no. 003770)^38^ were purchased from The Jackson Laboratory. *Tac1*-*tagRFP*-*2a*-*TVA* mouse line was described previously^56^, and the *Avil*-*Flp* line^57^ was a gift from D. Ginty. We also generated a new *Rosa*-*Cag*-*LSL*-*soma*-*jGCaMP8s* line used in crosses with *Sst*-*Cre*, an *Avil*-*LSL*-*2A*-*TeNT* line and a *Rosa*-*CAG*-*FSF*-*LSL*-*KORD* line using CRISPR–Cas9-mediated recombination^56^. Male and female mice were used in all experiments and given ad libitum access to standard laboratory chow and water. The mice were housed in a controlled environment (23 °C and 50% humidity with a 12-h light–dark cycle); no statistical methods were used to predetermine sample size.

### Injection of adeno-associated virus in mouse pups and CreERT2 induction

Left intracerebroventricular injection of 1 μl of AAV9-*Cag*-*Cre* virus (2 × 10^12^ to 2 × 10^13^ virions ml^−1^; catalogue no. CV17187-AAV9; Vigene) or AAV9-*CMV*-*Cre* virus (more than 1 × 10^13^ virions ml^−1^; catalogue no. 105537-AAV9; Addgene) in 1- to 3-day-old mouse pups containing Ai95 was used to achieve stochastic expression of GCaMP in neurons of the trigeminal ganglion as described previously^42^. To induce CreERT2 recombination, tamoxifen (Sigma-Aldrich) was dissolved in corn oil at a concentration of 20 mg ml^−1^ at 37 °C overnight. *Mrgprd*-*CreERT2* mice were injected intraperitoneally at a dosage of 75 mg tamoxifen per kg of body weight using an insulin syringe at least a week before calcium imaging.

### In vivo calcium imaging

For fluorescent calcium imaging of the trigeminal neurons, adult (over 8 weeks old) animals were subjected to one of three experimental regimes. Experimental regime A assessed both mechanical and temperature responses (Figs. 1 and 2 and Extended Data Figs. 1–5). The animals were anaesthetized with isoflurane and surgically prepared for optical access to the trigeminal ganglion as described previously^19^. The hairy skin of the cheek was mechanically stimulated using a series of manually delivered brushes with a cotton-tipped applicator and pinches with surgical forceps as described previously^3^. For improved temperature transfer, the mouse cheek was treated for less than 300 s with a depilatory cream (Veet) using a cotton-tip applicator. An eye ointment was applied to prevent damage to the cornea and conjunctiva, fur was removed, the cheek was washed at least three times with saline and dried with Kimwipes and a custom-built 5 mm^2^ Peltier probe (TCS2; QST.Lab) was applied directly to the skin for thermal stimulation. Peltier application did not induce long-term activity of trigeminal neurons, and LTMRs did not respond to heating (Fig. 1), ruling out major confounding effects related to mechanical stimulation by the probe. The skin was held at 30 °C for baseline non-stimulated activity measurements; temperature stimulation at 37, 39, 42, 45 and 50 °C was for 4 s.

Experimental regime B assessed the mechanical sensitization after inflammation (Fig. 4 and Extended Data Fig. 7). Baseline mechanical stimulation was as in experimental regime A. Inflammation of the cheek was then induced using subdermal injection of 20–30 μl, 0.5 mM PGE2 (Sigma-Aldrich) to three sites. After 10 min of incubation, inflammation was confirmed and mechanical stimulation was repeated.

**Extended Data Fig. 10:**
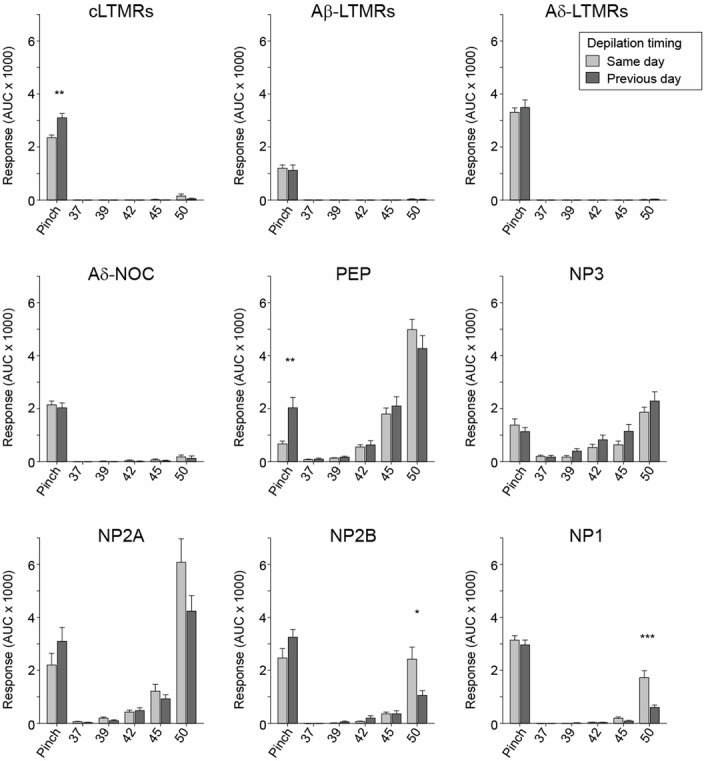
Similar response profiles in mice with different depilation timing. We used three different populations of mice during this study because we needed the fur intact to assess brushing and depilated skin for temperature series. Data in Figs. 1 and 2 were obtained from mice where the fur was removed acutely (during the functional imaging after mechanical stimulation had been performed). Data in other figures either used no fur removal (brush studies) or depilation was the previous day (approx. 24 h before recording) to minimize potential irritation from the chemical fur removal needed for temperature series. Note studies of inflammation were all carried out without acute fur removal. Response magnitudes (AUC mean ± s.e.m.) for same day (pale grey) and previous day (dark grey) depilation regimes for the different cell classes indicate only minimal differences in sensitivity; p < 0.05, *; p < 0.01, **; p < 0.001, ***. For details of statistical tests and numbers of mice and cells see Supplementary Information, Tables 1 and 2.

Experimental regime C assessed the thermal sensitization after inflammation (Figs. 3 and 5 and Extended Data Figs. 5 and 6). Mouse cheek depilation was carried out the day before functional imaging. Skin was subjected to a series of pinches and thermal stimulation as in experimental regime A. PGE2 or LY344864 (20–30 μl; 5 mg ml^−1^; MilliporeSigma) was injected as in experimental regime B, and stimulation was repeated. To evaluate consistency, responses to common stimuli were compared between experimental regimes A and C (Extended Data Fig. 10).

The functional activity of DRG neurons in the L5 and L6 ganglia was determined using fluorescent calcium imaging. PGE2 and LY344864 were injected as a single injection (volumes and concentrations as above) into the plantar hind paw. Mechanical stimulation was also applied to the plantar surface, which was held at 30 °C for the unstimulated recordings. For *Mrgprb4*-tdT-*2a*-*Cre* mice, the hairy skin of the leg was stimulated as NP2B neurons selectively innervated hairy skin^4^.

Calcium imaging was performed as described previously^3,58^ using a custom-built epifluorescence Cerna microscope (Thorlabs) and a pco.panda 4.2 bi CMOS camera; 40-s recording episodes were acquired at 5 Hz. For each experiment requiring post hoc ISH, either red fluorescent tagRFP images were collected or the trigeminal ganglion was briefly superfused with 500 μl of 1 M KCl to activate and visualize all GCaMP-expressing neurons after the experiment to provide alignment guide-posts. In vivo images were aligned and processed as described previously^3^.

### Spatial activity maps and analysis of fluorescence dynamics

Spatial activity maps and regions of interest (ROI) were generated as described previously^3^. In brief, activity induced by repetitive mechanical stimulation was visualized as standard deviation over time for each pixel. Heat-induced activity was visualized by subtracting the mean fluorescence before stimulation from the mean fluorescence during stimulation. ROI were manually extracted using the ‘Cell Magic Wand’ plugin in ImageJ. Overlapping cell ROI that were contaminated by each other’s responses were excluded from the analysis while blind to transcriptomic information. Relative change in GCaMP fluorescence was calculated as Δ*F*/*F* (%) for each cell, and potential contaminant signal from the underlying out-of-focus tissue and neighbouring cells was removed by subtracting the fluorescence of a doughnut-shaped area surrounding each cell using a custom MATLAB script^42^. Cell category-specific activity maps were generated by overlaying a category-specific mask over the activity map of the cognate stimuli (heat for heat-specific and polymodal cells; brush and pinch for mechano-specific cells).

### Whole-mount ISH of trigeminal ganglia

Whole-mount ISH of trigeminal ganglia after in vivo imaging and of tissue sections was performed as described previously^3^ using combinations of the following hybridization chain reaction probes (Molecular Instruments): *Trpm8* (GenBank NM_134252; full length), *S100b* (NM_009115; full length), *Fxyd2* (NM_007503; full length), *Scn10a* (NM_001205321; coding sequence), *Calca* (NM_007587; full length), *Trpv1* (NM_001001445; full length), *Tmem233* (NM_001101546; full length), *Mrgprd* (NM_203490; full length), *Nppb* (NM_008726; full length), *Sst* (NM_009215; full length), *Mlc1*(NM_133241; full length), *tagRFP*-*TVA*, tdT and *EGFP* (which detects GCaMP expression). Two-dimensional dorsal views of the surface of whole-mount ganglia were collapsed by maximum intensity projection from confocal *Z* stacks with 10-μm intervals to capture the convex surface of the ganglion.

### Aligning whole-mount ISH images to in vivo recordings

Alignment of whole-mount ISH images to in vivo fluorescent images was performed as described previously^3^ using either tagRFP-positive guide-post cells in *Tac1*-*tagRFP*/*TVA* animals or GCaMP-expressing cells stimulated with high K^+^ directly applied to the trigeminal ganglion. In brief, multi-channel two-dimensional ISH images were crudely aligned to in vivo fluorescence by scaled rotation using the TurboReg plugin and a custom macro in ImageJ/Fiji. Guide-post cells were then manually matched to their in vivo fluorescent counterparts using a custom ImageJ macro that identified coordinate pairs for each guide-post. The ISH image was morphed to match its in vivo counterpart using these coordinates with a custom Python script that builds on the OpenCV library^3^. Several rounds of ISH were aligned to each other using probes labelling partially overlapping sets of cells in both rounds to provide guide-posts for morphing. As shown previously^3^, this type of image alignment does not produce a pixel-to-pixel match but accurately identifies ISH-positive cells that respond functionally (Extended Data Fig. 2).

### Analysis of gene expression and transcriptomic classification

Cell ROI (responding cells) were manually analysed for expression (negative, weak or strong) of every gene with diagnostic ISH data^3^ (Extended Data Fig. 2c). Binary expression patterns were decoded into transcriptomic cell classes using the rules outlined in Extended Data Fig. 2b and Supplementary Table 3, which also explains how transcriptomic class nomenclature^10^ is related to other classification schemes.

Single-cell sequencing data from DRG^28^ were obtained from GEO Series GSE254789 and analysed with Seurat v.5 in RStudio. Cells with less than 800 expressed genes or more than 5% of mitochondrial transcripts were excluded, and datasets were combined using canonical correlation analysis integration after principal component analysis reduction to 30 components. Neuronal and non-neuronal cell clusters were identified in UMAP by analysing the expression of *Snap25*, *Mbp*, *Apoe*, *Qk*, *Pecam1*, *Slc17a7* and *Slc17a6*. Doublets were identified using DoubletFinder v.3, and doublets and non-neuronal cells were removed from the dataset. After neurons were renormalized, reduced to 40 principal components and reintegrated, neuronal clusters were calculated using the Louvain algorithm with a resolution of 0.2 and identified/combined on the basis of the genes shown in Extended Data Fig. 2.

### Behavioural assessment of allodynia

For behavioural experiments, groups of adult C57Bl/6, *Trpv1*-*Cre*::*Avil*-*LSL*-TeNT and control littermates were tested. RNAscope ISH of fresh frozen sections of DRG^12^ (Advanced Cell Diagnostics) was used to examine the extent and selectivity of TeNT recombination. The cheek or plantar surface of the hind paw was injected with PGE2, LY344864 or phosphate-buffered saline (PBS) as described above. Mice (male and female; more than 8 weeks old) were habituated to the testing chambers for at least two sessions in the days preceding the behavioural tests. When mice were used for more than one experiment, they were allowed at least 7 days to recover between tests. The experimenter was blinded to the genotype of the animals.

For the data shown in Fig. 5a,e, mechanical thresholds (50% withdrawal threshold) were determined using von Frey stimulation by the simplified up–down method^59^ at multiple time points up to 2 h after injection of PGE2 or LY344864. The experimenter was blinded to the genotypes of the mice. For Fig. 5f, mechanical threshold (60% withdrawal threshold; three responses in five trials) was determined using von Frey stimulation by a standard up–down method at baseline and a single time point 15–30 min after intraperitoneal injection of vehicle (dimethylsulfoxide) or Salvinorin B (Hello Bio; 10 mg ml^−1^; 10 mg kg^−1^) and paw injection of LY344864 (as described above). The experimenter was blinded to the injected compound. The mice were tested twice (opposite paws; 6 days apart), with four receiving Salvinorin B in the first test and the other three in the second round. The assignment of mice to the two groups was pseudorandom. Brush allodynia was determined in the same behavioural apparatus by stimulating C57Bl/6 mice. As shown in Extended Data Fig. 9a, 20 brushes were delivered using a paint brush (7950-5 Round; KINGART) once every minute before and 10–30 min after paw injection. A response was counted as any withdrawal from the stimulation. As shown in Extended Data Fig. 9b, a single brush with a fluffed cotton swab was delivered at baseline and at each time point after paw injection of PBS or LY344864. Pain-like behaviours (repetitive or extended lifting and guarding) and brief response to brushing were scored. The experimenter was blinded to the injected compound; thus, mice were randomly assigned to groups.

Spontaneous pain behaviours following the injection of 500 µM PGE2 (in 20 µl of PBS) or PBS alone into the hind paw or cheek were recorded and scored offline using BORIS^60^ by an observer blinded to the genotype and/or the compounds used. In mice injected in the hind paw, licking of the injected paw was scored for 15 min and quantified for a 10-min period, starting 5 min after injection to match the development of inflammation^37^. For cheek injection blinding, the animals were randomly assigned to groups. Following injections, the mice were placed in cylindrical plexiglass chambers surrounded by mirrors. All face-directed behaviours and periods of inactivity greater than 1 s were scored for the first 15 min after injection and quantified for the same 10-min period used for the paw. Although inactivity may represent freezing-like behaviour (Supplementary Video 3), it may also represent sitting or sleeping. The single mouse injected with PBS that displayed considerable inactivity did not appear to enter a freeze-like state, whereas the majority of the PGE2-injected mice did. To avoid judgement errors in scoring at the resolution of the videos, inactivity was analysed without trying to assess whether the mouse was in distress. The right cheek was partially depilated 2 days before behavioural recording to aid injection.

### Quantification and statistical analysis

The numbers of animals and responding cells that were tested for each transcriptomic class are listed in Supplementary Table 1. All quantification and statistical analyses were performed using Python v.3.8, Pandas v.1.1.3, Numpy v.1.19.2 and Scipy v.1.5.2.

Spontaneous activity was detected as peaks in Δ*F*/*F* traces with a minimum prominence of 4% Δ*F*/*F*, a minimum absolute peak of 4% Δ*F*/*F* and a minimum interpeak interval of 0.6 s using the Scipy find_peaks function. The amplitude of an event was calculated as the difference between peak height and its preceding minimum. Spontaneous activity was quantified over multiple time windows when the cheek or paw was held at 30 °C (105 s for trigeminal neurons and 40 s for DRG) by summing event amplitudes.

Temperature-induced responses were identified as peaks with a minimum prominence of 5% Δ*F*/*F*, a minimum interpeak interval of 0.6 s and an onset during the temperature stimulation window. The end of a response was defined as the time point when signal dropped below 10% peak height. The area under the Δ*F*/*F* curve from onset to end of the response was used to quantify response to temperature. A cell was considered responsive to a temperature stimulus if AUC exceeded a defined minimum (corresponding to a mean of 3.5% Δ*F*/*F* over the 4-s stimulation window). A cell was mechanosensitive if its peak amplitude exceeded 15% Δ*F*/*F* during the stimulus application window. Cells were polymodal if the ratio between the mechanical and temperature stimuli was smaller than 5:1 and larger than 1:5.

Quantification of responses for a given stimulus varies according to the choice of cells included in the analysis. For example, cells may have spontaneous activity but not respond to mechanical or thermal stimulation of the cheek. For consistency and to allow comparison between figures, we report the response magnitudes (and numbers) of cheek-innervating neurons, that is, cells that responded to any stimulus applied to the cheek.

To distinguish brush responses from spontaneous activity after chemical induction, individual time-locked brush responses within a 1-s window of stimulus application were identified as peaks with a minimum prominence of 5% Δ*F*/*F* and a minimum absolute peak height of 20% Δ*F*/*F*. Bona fide brush cells were identified as cells that responded to at least 50% of brushes, which cover largely but not completely overlapping fields of the cheek.

Percentages of transcriptomic cell classes contributing to a functional cell category were calculated as described previously^3^ by dividing the number of responding cells positive for a given class by the number of responding cells that were tested with ISH probes for that class. Because not all classes were tested in all individual animals, the summed percentages do not necessarily add up to 100%. To display proportions in stacked bar graphs, the percentages were further normalized to 100% in these graphs.

The effects of PGE2 injection were analysed using two-tailed paired Student’s *t*-test. Attenuation of heat responses by *Trpv1* knockout was analysed using one-tailed Welch’s *t*-test (allowing for unequal variances between different conditions). The effects of *Trpv1* knockout on spontaneous activity were analysed using two-tailed Welch’s *t*-test.

Holm–Šidák correction was applied to all statistical tests to adjust for multiple comparisons when investigating several transcriptomic classes. One-tailed Wilcoxon signed-rank tests were used for von Frey thresholds and brush-induced behaviour when comparing time points after allodynia induction to paired baseline values. In Extended Data Fig. 9b, data were pooled across time points after injection and compared between experimental groups with a chi-squared test. All other comparisons between behavioural groups used the Mann–Whitney *U*-test. Detailed statistical information is provided in Supplementary Table 2.

### Reporting summary

Further information on research design is available in the Nature Portfolio Reporting Summary linked to this article.

## Online content

Any methods, additional references, Nature Portfolio reporting summaries, source data, extended data, supplementary information, acknowledgements, peer review information; details of author contributions and competing interests; and statements of data and code availability are available at 10.1038/s41586-025-08875-6.

## Supplementary information

Supplementary Table 1 **Experimental regimes and numbers of animals and cells**. Sample size information.

Reporting Summary

Supplementary Table 2 **Statistical reporting**. Statistical tests and *P* values.

Supplementary Table 3 **Logic for ISH-based classification**. Details of cell classification strategies.

Supplementary Video 1 **Spontaneous activity in trigeminal neurons before and after PGE2-induced inflammation**. Videos (2.4 times recording speed) of ganglion imaging (Δ*F*/*F*) for the same cheek-responsive field of the trigeminal ganglion at baseline (left) and during PGE2-induced continuing inflammation (right). The cheek was held at 30 °C for the duration of the recordings.

Supplementary Video 2 **Behavioural response of a mouse minutes after cheek injection of PBS**. Mouse injected in the right cheek with PBS exhibiting a typical range of behaviours.

Supplementary Video 3 **Behavioural response of a mouse minutes after cheek injection of PGE2**. Mouse injected in the right cheek with PGE2; note that the mouse remained immobile for extended periods (a behaviour rarely seen after PBS injection), adopted a hunched posture and exhibited noticeable tremors.

Supplementary Video 4 **A mouse exhibiting incomplete face-directed behavioural responses after cheek injection of PGE2**. Mouse injected in the right cheek with PGE2 showing unusual behavioural responses approximately 4 min after injection. Note that the mouse repeatedly raised a paw towards the injected cheek but failed to touch the skin.

## Data Availability

Data needed to interpret, verify and extend the research (calcium traces, ISH annotations and behavioural data) are available at Zenodo (10.5281/zenodo.14907827)^61^. Data from GEO Series GSE254789 were also analysed.

## Code availability

Custom code for analysis is available at Zenodo (10.5281/zenodo.14907827)^61^.
