# Supplementary material for: A distributed coding logic for thermosensation and inflammatory pain
Source: Nature. 2025 Apr 23;642(8069):1016–23. doi: 10.1038/s41586-025-08875-6 (PMC12222022; doi:10.1038/s41586-025-08875-6)
Supplement: Supplementary file 2 — Reporting Summary [file 41586_2025_8875_MOESM2_ESM.pdf]

nature portfolio

Corresponding author(s): Ryba & Chesler

Last updated by author(s): 2/25/2025

Reporting Summary

Nature Portfolio wishes to improve the reproducibility of the work that we publish. This form provides structure for consistency and transparency in reporting. For further information on Nature Portfolio policies, see our Editorial Policies and the Editorial Policy Checklist.

Statistics

For all statistical analyses, confirm that the following items are present in the figure legend, table legend, main text, or Methods section.

n/a	Confirmed	
☐	☒	The exact sample size (*n*) for each experimental group/condition, given as a discrete number and unit of measurement
☐	☒	A statement on whether measurements were taken from distinct samples or whether the same sample was measured repeatedly
☐	☒	The statistical test(s) used AND whether they are one- or two-sided<br>*Only common tests should be described solely by name; describe more complex techniques in the Methods section.*
☐	☒	A description of all covariates tested
☐	☒	A description of any assumptions or corrections, such as tests of normality and adjustment for multiple comparisons
☐	☒	A full description of the statistical parameters including central tendency (e.g. means) or other basic estimates (e.g. regression coefficient) AND variation (e.g. standard deviation) or associated estimates of uncertainty (e.g. confidence intervals)
☐	☒	For null hypothesis testing, the test statistic (e.g. F, t, r) with confidence intervals, effect sizes, degrees of freedom and P value noted<br>*Give P values as exact values whenever suitable.*
☒	☐	For Bayesian analysis, information on the choice of priors and Markov chain Monte Carlo settings
☒	☐	For hierarchical and complex designs, identification of the appropriate level for tests and full reporting of outcomes
☒	☐	Estimates of effect sizes (e.g. Cohen's *d*, Pearson's *r*), indicating how they were calculated

Our web collection on statistics for biologists contains articles on many of the points above.

Software and code

Policy information about availability of computer code

Data collection	Excelitas PCO Camera Control Software; pCLAMP-11; Zeiss ZEN Black
Data analysis	ImageJ-1.53t; Custom MATLAB and Python scripts from github.com/lars-von-buchholtz/warping; R-script & Jupyter notebook from DOI:10.5281/zenodo.14907827; R version 4.4.0; Seurat version 5.1.0; https://github.com/chris-mcginnis-ucsf/DoubletFinder version 2.0.4; Python 3.8; Numpy 1.24.3; Pandas 1.4.4; Matplotlib 3.7.2; Seaborn 0.13.2; Scikit-image 0.20.0; Statsmodels 0.14.0; Scipy 1.9.1

For manuscripts utilizing custom algorithms or software that are central to the research but not yet described in published literature, software must be made available to editors and reviewers. We strongly encourage code deposition in a community repository (e.g. GitHub). See the Nature Portfolio guidelines for submitting code & software for further information.

Data

Policy information about availability of data

All manuscripts must include a data availability statement. This statement should provide the following information, where applicable:

- Accession codes, unique identifiers, or web links for publicly available datasets
- A description of any restrictions on data availability
- For clinical datasets or third party data, please ensure that the statement adheres to our policy

Data needed to interpret, verify and extend the research (calcium-traces, ISH annotations and behavioral data) have been deposited at Zenodo https://doi.org/10.5281/zenodo.14907827. Data from GEO, Series GSE254789 were also analyzed.

Research involving human participants, their data, or biological material

Policy information about studies with human participants or human data. See also policy information about sex, gender (identity/presentation), and sexual orientation and race, ethnicity and racism.

Reporting on sex and gender	N/A
Reporting on race, ethnicity, or other socially relevant groupings	N/A
Population characteristics	N/A
Recruitment	N/A
Ethics oversight	N/A

Note that full information on the approval of the study protocol must also be provided in the manuscript.

Field-specific reporting

Please select the one below that is the best fit for your research. If you are not sure, read the appropriate sections before making your selection.

☒ Life sciences ☐ Behavioural & social sciences ☐ Ecological, evolutionary & environmental sciences

For a reference copy of the document with all sections, see nature.com/documents/nr-reporting-summary-flat.pdf

Life sciences study design

All studies must disclose on these points even when the disclosure is negative.

Sample size	No explicit sample size estimates were made. Where applicable, sample sizes were estimated based on experience and previously published studies refs 3, 58 & 59.
Data exclusions	Data not included: Results from a number of mice which either died before a full stimulation protocol was performed or for which the ISH after functional imaging failed. Cells that were not in focus or had extensive overlap in response and cells that were not responsive to cheek stimulation were excluded. For mice not tested for inflammation: cells that had spontaneous activity were also excluded. In addition, responsive cells that could not be mapped to ISH based on GCaMP expression and cells that could not be unambiguously mapped to cell class were excluded from the full analysis. Very few "Cool" neurons were identified as innervating the cheek; 6 such Trpm8 expressing "Cool" neurons from the entire dataset are reported in the supplementary tables but have been excluded from the main figures.
Replication	Data were replicated across animals and experiments with full details in the figure legends and in Supplementary Information Table 1. For representative images shown experiments were replicated in at least 3 mice.
Randomization	Randomization was used for behavioral experiments where appropriate (e.g., mice were randomly assigned to groups and where injected with two compounds, the order was pseudo-random); randomization is not appropriate for other experiments (ISH and Ca-imaging)
Blinding	Experimenter performing behavioral studies was blinded to mouse genotype and/or compound injected. Experimenter annotating ISH data was blind to functional response of the cells.

Reporting for specific materials, systems and methods

We require information from authors about some types of materials, experimental systems and methods used in many studies. Here, indicate whether each material, system or method listed is relevant to your study. If you are not sure if a list item applies to your research, read the appropriate section before selecting a response.

Materials & experimental systems

n/a	Involved in the study	
☒	☐	Antibodies
☒	☐	Eukaryotic cell lines
☒	☐	Palaeontology and archaeology
☐	☒	Animals and other organisms
☒	☐	Clinical data
☒	☐	Dual use research of concern
☒	☐	Plants

Methods

n/a	Involved in the study	
☒	☐	ChIP-seq
☒	☐	Flow cytometry
☒	☐	MRI-based neuroimaging

Animals and other research organisms

Policy information about studies involving animals; ARRIVE guidelines recommended for reporting animal research, and Sex and Gender in Research

Laboratory animals	Mouse (Mus musculus) C57Bl/6 and mixed background (C57Bl/6 -FVB/N - C3H) strains
Wild animals	none used
Reporting on sex	All experiments used male and female mice
Field-collected samples	none used
Ethics oversight	The NIDCR and NINDS Animal Care and Use Committees approved experiments performed in these studies. Protocol numbers: NIDCR 23-1127, 21-1069, NINDS 1365 and 1369

Note that full information on the approval of the study protocol must also be provided in the manuscript.

Plants

Seed stocks	N/A
Novel plant genotypes	N/A
Authentication	N/A
