# Supplementary material for: A distributed coding logic for thermosensation and inflammatory pain
Source: Nature. 2025 Apr 23;642(8069):1016–23. doi: 10.1038/s41586-025-08875-6 (PMC12222022; doi:10.1038/s41586-025-08875-6)
Supplement: Supplementary file 4 — Logic for ISH-based classification. Details of cell classification strategies. [file 41586_2025_8875_MOESM4_ESM.pdf]

Supplementary Information, Table 3. Logic for ISH-based classification

**Cell Class**	**Alias**[3]	**Gene expression combinations used for class assignment**
Cool	C1/2	*Trpm8*
cLTMR	C3	*Fxyd2* ∧ ¬*Tmem233* ∧ ¬*S100b*
Aβ-LTMR	C4	*S100b* ∧ ¬*Fxyd2* ∧ ¬Scn10a
Aδ-LTMR	C5	*S100b* ∧ *Fxyd2*
Aδ-NOC	C6	*S100b* ∧ (*Scn10a* ∨ *Calca*)
PEP	C7-10	(*Trpv1* ∨ *Calca*) ∧ ¬*Tmem233* ∧ ¬*S100b* ∧ ¬*Fxyd2*
NP3	C11	*Nppb* ∨ *Sst*
NP2A	C12	*Tmem233* ∧ ¬*S100b* ∧ ¬*Mrgprd* ∧ ¬(*Nppb* ∨ *Sst)* ∧ strong *Calca*
NP2B	C12	*Tmem233* ∧ ¬*S100b* ∧ ¬*Mrgprd* ∧ ¬(*Nppb* ∨ *Sst)* ∧ ¬(strong *Calca*)
NP1	C13	*Mrgprd*

¬X = logical NOT (complement of X)

X ∧ *Y* = logical AND (intersection of X and Y)

X ∨ *Y* = logical OR (union of X and Y)
